# Clinical characteristics and outcomes of overt gastrointestinal bleeding in children undergoing haploidentical hematopoietic stem cell transplantation: a single-center retrospective analysis

**DOI:** 10.1186/s12887-024-04950-5

**Published:** 2024-07-27

**Authors:** Shanshan Qi, Lannan Zhang, Zhi Chen, Zhuo Wang, Lili Ding, Yu Du, Hao Xiong

**Affiliations:** 1 Laboratory of Pediatric Hematology, Wuhan Children’s Hospital, Tongji Medical College, Huazhong University of Science and Technology, 100 Hong Kong Road, Wuhan, 430015 China; 2 Department of Hematology, Wuhan Children’s Hospital, Tongji Medical College, Huazhong University of Science and Technology, 100 Hong Kong Road, Wuhan, 430015 China

**Keywords:** Children, Haploidentical hematopoietic stem cell transplantation, Overt gastrointestinal bleeding, Risk factors

## Abstract

**Background:**

Overt gastrointestinal bleeding (GIB) is a potentially serious and life-threatening condition in patients undergoing allogeneic hematopoietic stem cell transplantation (allo-HSCT). However, relatively little information is available regarding overt GIB in children.

**Objectives:**

To assess the prevalence, clinical patterns, and outcomes of overt GIB in children undergoing haploidentical hematopoietic stem cell transplantation (haplo-HSCT).

**Methods:**

A total of 123 consecutive patients with malignant or non-malignant blood disorders who received haplo-HSCT were reviewed in our hospital between October 2017 and October 2022. Overt GIB was determined as hematemesis, melena or hematochezia. Continuous variables were compared by Mann Whitney U test. Categorical parameters were compared by the χ^2^ test or Fisher’s exact test. Kaplan-Meier curves and log-rank tests were used to assess overall survival (OS), non-relapse mortality (NRM) and relapse. Univariate and multivariate analyses were performed to identify potential risk factors of overt GIB development.

**Results:**

The median follow-up was 26.3 (range,1.7–74.8) months. Overt GIB occurred in 31 patients (25.2% incidence), with a median time elapsed after haplo-HSCT of 376 days (range, 58–1275 days). Compared with the non-GIB group, patients with overt GIB had reduced OS and increased NRM. In multivariate analysis, grade III–IV gut acute graft versus-host disease (aGvHD), thrombotic microangiopathy (TMA) and cytomegalovirus (CMV) viremia were significant risk factors for the occurrence of overt GIB after haplo-HSCT.

**Conclusions:**

Overt GIB is a frequent complication after haplo-HSCT in pediatric patients, and associated with worse survival. Grade III–IV gut aGvHD, TMA and CMV viremia were associated with its development.

**Supplementary Information:**

The online version contains supplementary material available at 10.1186/s12887-024-04950-5.

## Introduction

Allogeneic hematopoietic stem cell transplantation (allo-HSCT) has been increasingly used to treat a wide range of malignant and non-malignant disorders in children [1–3]. However, its use has been limited by a lack of human leukocyte antigen (HLA)-matched donors. Only 50% of children who require an allo-HSCT will have an HLA-matched donor, and this percentage falls to less than 20% for some ethnic minority populations [4–6]. For patients without an HLA-matched donor, haploidentical hematopoietic stem cell transplantation (haplo-HSCT) is therefore an important alternative option [7, 8]. Along with the wide use of anti-thymocyte globulin (ATG)-based or post-transplant cyclophosphamide (PTCy)-based regimens, haplo-HSCT is feasible and achieves good results for a range of hematological diseases [9, 10].

With the extended application of haplo-HSCT, a variety of complications have become increasingly recognized. Gastrointestinal disorders are common after HSCT and usually caused by chemotherapeutic drugs, opportunistic infections, acute graft-versus-host disease (aGvHD) or coagulation abnormalities [11–13]. Gastrointestinal bleeding (GIB) is one of the most common manifestations of gastrointestinal disorders and is associated with significant mortality [14, 15]. However, the majority of literature on HSCT-associated GIB studies describe adult patients [16, 17], with relatively little information available for pediatric GIB, especially in a haplo-HSCT population.

Herein, we reviewed the incidence, causes, clinical features, outcomes, and risk factors for GIB in pediatric patients receiving haplo-HSCT in a single unit.

## Methods

### Patients

We retrospectively reviewed the medical records of patients received haplo-HSCT at Wuhan Children’s Hospital between October 2017 to October 2022. Overall, 123 patients underwent haplo-HSCT. Based on medical records, 31 patients were diagnosed with overt GIB. This study was approved by the Medical Ethics Committee of Wuhan Children’s Hospital, Tongji Medical College, Huazhong University of Science and Technology.

### Preparative regimens, GvHD prophylaxis and stem cell sources

For patients diagnosed with acute myeloid leukemia or myelodysplastic syndromes who underwent haplo-HSCT, the conditioning regimens included cyclophosphamide (10 mg/kg/day on days − 8 and − 7), intravenous fludarabine (30 mg/m^2^/day from day − 6 to day − 2), oral semustine (250 mg/m^2^ on day − 6), and intravenous busulfan (1.1 mg/kg/day from day − 5 to day − 2). PTCy-based prophylactic regimens were used for GvHD prophylaxis. Cyclosporine, rabbit ATG, rituximab, mycophenolate mofetil and cyclosporine A were included. As described recently [18], donor lymphocyte infusion (DLI) was given depending on the clinical status.

For patients with acute lymphocytic leukemia, the conditioning regimens included total marrow irradiation (4 Gy/d on day − 10 and day − 9), etoposide (20 mg/kg/d on day − 6 and − 5), rabbit ATG (10 mg/kg from day − 4 to day − 2) and cyclophosphamide (60 mg/kg/d on day − 3 and − 2). For non-malignant disorders in our study, the conditioning regimens included cyclophosphamide (120 mg/kg from day − 8 to day − 7), intravenous fludarabine (40 mg/m^2^/day from day − 6 to day − 3), intravenous busulfan (1.1 mg/kg/day on day − 6 and day − 5) and rabbit ATG (10 mg/kg from day − 4 to day − 2). GvHD prophylaxis consisted of mycophenolate mofetil, short-term methotrexate and cyclosporine.

Acute GVHD (aGvHD) and chronic GVHD (cGvHD) were diagnosed and graded according to the established guidelines and criteria [19, 20]. Patients were evaluated for aGVHD occurring within 100 days after DLI.

All the patients received granulocyte colony-stimulating factor modified peripheral blood stem cell transplantation grafts.

### Definitions

In terms of clinical outcomes, overall survival (OS) was defined as the interval from HSCT to death irrespective of disease state. Non-relapse mortality (NRM) was defined as death without previous relapse. Cytomegalovirus (CMV) viremia was defined as serum CMV-DNA was more than 1000 copies/mL. Diagnosis of intestinal infections was made based on gastrointestinal symptoms and laboratory examination results. Overt GIB was defined as the presence of hematemesis, melena or hematochezia. Severe GIB was defined as either (1) overt GIB resulting in hemorrhagic shock or (2) overt GIB leading to a decrease in hemoglobin of ≥ 2 g/dL or at least a 20% fall in hematocrit, and transfusion at least two units of packed red blood cells.

### Attribution of GIB causes

The cause of GIB was determined by clinical review of endoscopic, histological and microbiological data. Gut GvHD was recognized as the sole cause of bleeding when the clinical and histological appearances were typical, and cultures or immunohistochemistry stains for viruses and fungi were negative. When there was evidence of gut GvHD and infection, both were regarded as the causes of bleeding [16, 21].

Other causes of bleeding, such as gastritis and ulcers, were based on clinical and endoscopic criteria.

### Statistical analysis

Continuous variables in our study were presented as medians (ranges) and were compared using the Mann–Whitney test. Categorical variables were compared using Pearson’s Chi-square test or Fisher’s exact test. Logistic regression was used to examine correlations. OS, NRM and relapse were assessed by the Kaplan–Meier method and compared using the log-rank test. *P*-values < 0.05 were considered to be significant. Data were analyzed using R software, version 4.1.0. and Prism version 8.4.3.

## Results

### Baseline patient characteristics

Overt GIB occurred in 31 out of 123 patients after haplo-HSCT, giving an incidence of 25.2%. The baseline characteristics of the two groups are shown in Table 1. More patients with GIB experienced grade III-IV gut aGvHD, thrombotic microangiopathy (TMA), intestinal infection and cytomegalovirus (CMV) viremia than patients in the control group. There were no differences between the two groups in the distribution of age, gender, underlying diseases, HLA mismatching, blood type compatibility, donor-recipient sex mismatching, engraftment time, preparative regimens, GvHD prophylaxis and CMV DNA.

**Table 1:** Baseline characteristics in patients with overt GIB and matched controls after haplo-HSCT

Variables	Overt GIB (n = 31)	Non-GIB (n = 92)	P value
Age at HSCT, yr (range)	5.5 (0.8–16.2)	5.8 (0.2–16.9)	0.7664
Gender (n, %)			
Male	16 (51.6%)	53 (57.6%)	0.561
Female	15 (48.4%)	39 (42.4%)	
Median follow-up time after HSCT, m (range)	22.5 (1.9–60.1)	29.5 (1.7–74.8)	0.2808
Underlying disease, n (%)			0.445
Aplastic anemia	6 (19.4%)	29 (31.5%)	
Acute myeloid leukemia	14 (45.2%)	32 (34.8%)	
Acute lymphoblastic leukemia	2 (6.5%)	11 (12.0%)	
Myelodysplastic syndromes	5 (16.1%)	8 (8.7%)	
Others*	4 (12.9%)	12 (13.0%)	
Conditioning, n (%)			0.182
CTX/Flu/Me-CCNU/Bu	19	39	
CTX/Flu/Bu/ATG	10	42	
TMI/VP-16/ATG/CTX	2	11	
GvHD prophylaxis, n (%)			0.068
PTCy-based regimens	19	39	
ATG-based regimens	12	53	
ABO mismatch, n (%)	17 (54.8%)	49 (53.3%)	0.713
Donor-recipient sex mismatch, n (%)	17 (54.8%)	45 (48.9%)	0.568
Human leukocyte antigen, n (%)			
5/10	16 (51.6%)	38 (41.3%)	0.722
6/10	6 (19.4%)	29 (31.5%)	
7/10	5 (16.1%)	12 (13.0%)	
8/10	2 (6.5%)	7 (7.6%)	
9/10	2 (6.5%)	6 (6.5%)	
MNC (×10^8^/kg, range)	18.9 (7.60–35.72)	16.12 (4.69–35.00)	0.2409
CD34^+^ (×10^6^/kg, range)	11.42 (4.0–24.0)	10.06 (3.34–21.98)	0.2175
Engraftment time, d (range)			
Neutrophil	15 (11–35)	14 (10–73)	0.0933
Platelet	16 (8–26)	15 (10–40)	0.322
Gut aGvHD, n (%)			< 0.0001
0	6 (19.4%)	51 (54.4%)	
I–II	14 (45.2%)	34 (37.0%)	
III–IV	11 (35.5%)	7 (7.6%)	< 0.0001
Extensive cGvHD, n (%)	5 (16.1%)	10 (10.9%)	0.439
Sinusoidal obstruction syndrome	4 (12.9%)	6 (6.5%)	0.269
Thrombotic microangiopathy	7 (22.6%)	4 (4.3%)	0.005
Disseminated intravascular coagulation	1 (3.2%)	2 (2.2%)	0.743
Acute kidney injury	1 (3.2%)	4 (4.3%)	0.784
Hemorrhagic cystitis	10 (32.3%)	21 (22.8%)	0.296
Intestinal infection	9 (29.0%)	8 (8.7%)	0.005
CMV viremia	24 (77.4%)	39 (42.4%)	0.001
CMV DNA (×10^3^copies/mL)	7.19 (1.28–489.0)	3.11 (1.11–38.5)	0.191

*Notes* *, Fanconi anemia, Hemophagocytic syndrome, Thalassemia, Wiskott-Aldrich syndrome, Severe combined immunodeficiency; aGvHD, acute graft versus host disease; ATG, anti-thymocyte globulin; Bu, busulfan; cGvHD, chronic graft versus host disease; CMV, cytomegalovirus; CTX, cyclophosphamide; GIB, gastrointestinal bleeding; Flu, fludarabine; HSCT, allogeneic hematopoietic stem cell transplantation; Me-CCNU, semustine; MNC, mononuclear cell; PTCy, posttransplant cyclophosphamide; TMI, total marrow irradiation; VP-16, etoposide

### GIB after haplo-HSCT

**Fig. 1:**
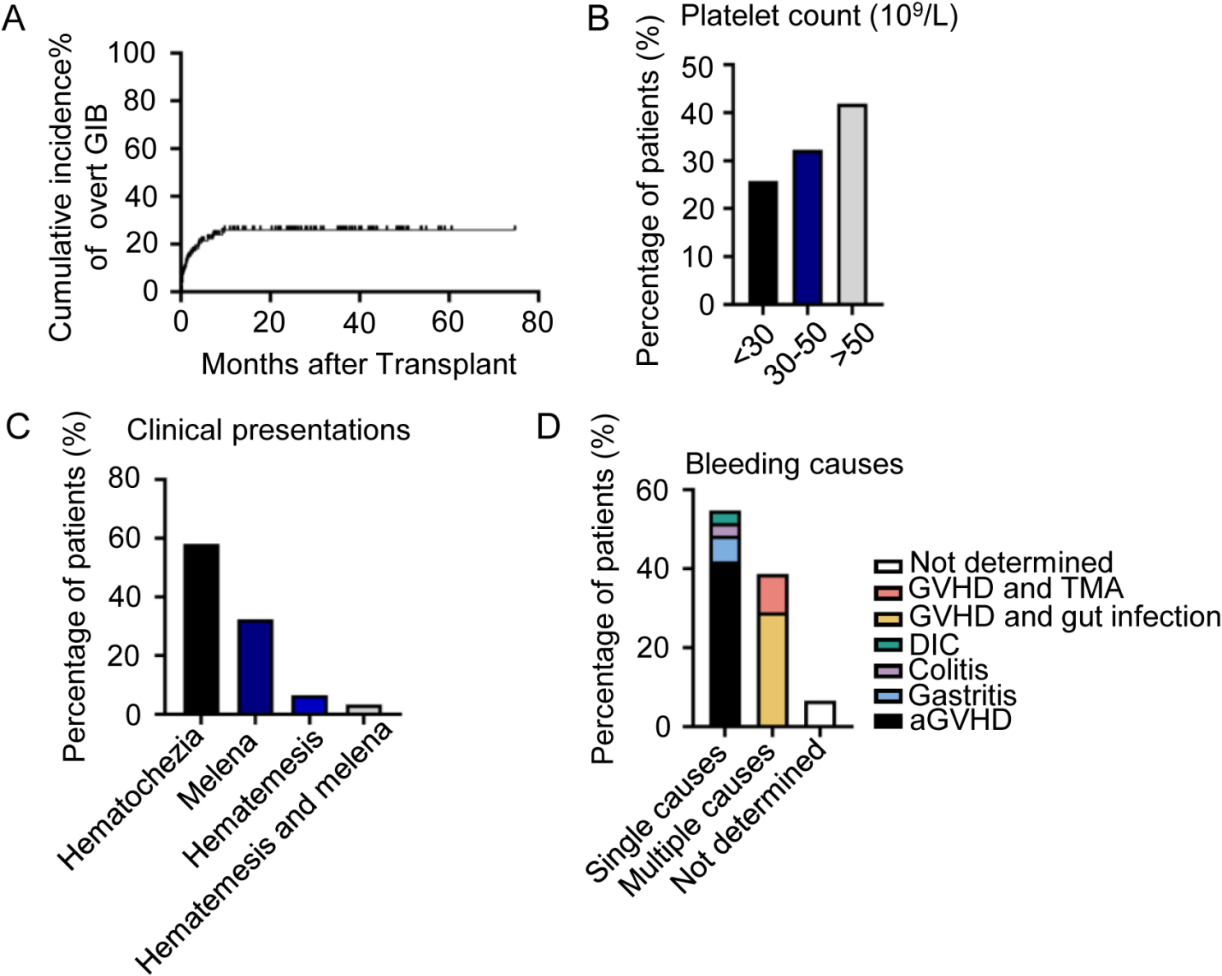
Characteristics of patients with overt GIB. (**A**) Incidence of GIB after haplo-HSCT. (**B**) Proportion of GIB patients with different platelet counts. (**C**) Proportion of GIB patients with different clinical presentations. (**D**) Proportion of GIB patients with different bleeding causes. aGVHD, acute graft versus host disease; DIC, disseminated intravascular coagulation; GIB, gastrointestinal bleeding; TMA, thrombotic microangiopathy

The median time after haplo-HSCT for the overt GIB event to occur was 376 days (range, 58 − 1,275 days) (Fig. 1.A and Supplemental Fig. 1.A). A total of 18 (18/31, 58.1%) patients had thrombocytopenia (platelet count < 50 × 10^9^/L) before GIB (Fig. 1.B), and 3 (3/31, 9.7%) patients required transfer to the medical intensive care unit for closer monitoring and management. The clinical presentations during GIB were variable. Of the 31 patients, 18 patients had hematochezia (58.1%), 10 patients had melena (32.3%), 2 patients had hematemesis (6.4%), and 1 patient (3.2%) presented with both hematemesis and melena (Fig. 1.C). The causes of GIB were diverse, with the most common single cause of bleeding being gut aGvHD (13/31, 41.9%), followed by gastritis (2/31, 6.5%), colitis (1/31, 3.2%) and disseminated intravascular coagulation (DIC, 1/31, 3.2%). Multiple bleeding causes were found in 12 patients (12/31, 38.7%): gut GvHD (8 aGvHD and one moderate cGvHD) and gut infection both contributed to the occurrence of GIB in 9 patients (9/31, 29%), and 3 patients bled due to gut aGvHD and TMA (3/31, 9.7%). The causes of bleeding were not determined in two patients (2/31, 6.5%) (Fig. 1.D). The grade and treatment of GvHD were shown in Supplemental Fig. 1.B and C.

### Severe GIB after haplo-HSCT

Among patients with overt GIB, five patients (5/31, 16.1%) experienced severe GIB and presented with hematochezia (Table 2). For severe GIB, two cases were patients with aplastic anemia, two patients had acute myeloid leukemia, and one patient had myelodysplastic syndromes. All of the patients with severe GIB had thrombocytopenia. Two of these five patients died during the hospitalization for severe GIB, with causes of death recorded as DIC and TMA, respectively. No deaths were directly attributable to the GIB.

**Table 2:** Characteristics of patients with severe GIB

Case	Sex	Age (yr)	Underlying disease	Donor	ABO mismatch	HLA	Occurrence time after HSCT (d)	Platelet count (10^9^/L)	GvHD Grade	Intestinal infection and pathogens	CMV viremia	Outcomes	Causes of death
1	Male	6.0	Aplastic anemia	Father	Yes	5/10	37	12	II aGVHD	No	No	Alive	/
2	Male	15.6	Aplastic anemia	Mother	No	5/10	279	7	Moderate cGVHD	Yes/CMV	Yes	Death	TMA
3	Male	1.6	Acute myeloid leukemia	Father	Yes	5/10	26	33	III aGVHD	Yes/Clostridium difficile	Yes	Alive	/
4	Female	15.0	Acute myeloid leukemia	Mother	Yes	5/10	294	36	IV aGVHD*	No	Yes	Death	DIC
5	Female	5.5	Myelodysplastic syndromes	Father	Yes	7/10	18	23	IV aGVHD	Yes/ Enteropathogenic Escherichia coli	Yes	Alive	/

*Notes* aGVHD, acute graft versus host disease; CMV, cytomegalovirus; cGVHD, chronic graft versus host disease; DIC, disseminated intravascular coagulation; TMA, thrombotic microangiopathy;*: Occurring within 100 days after donor lymphocyte infusion

### Outcomes

**Fig. 2:**
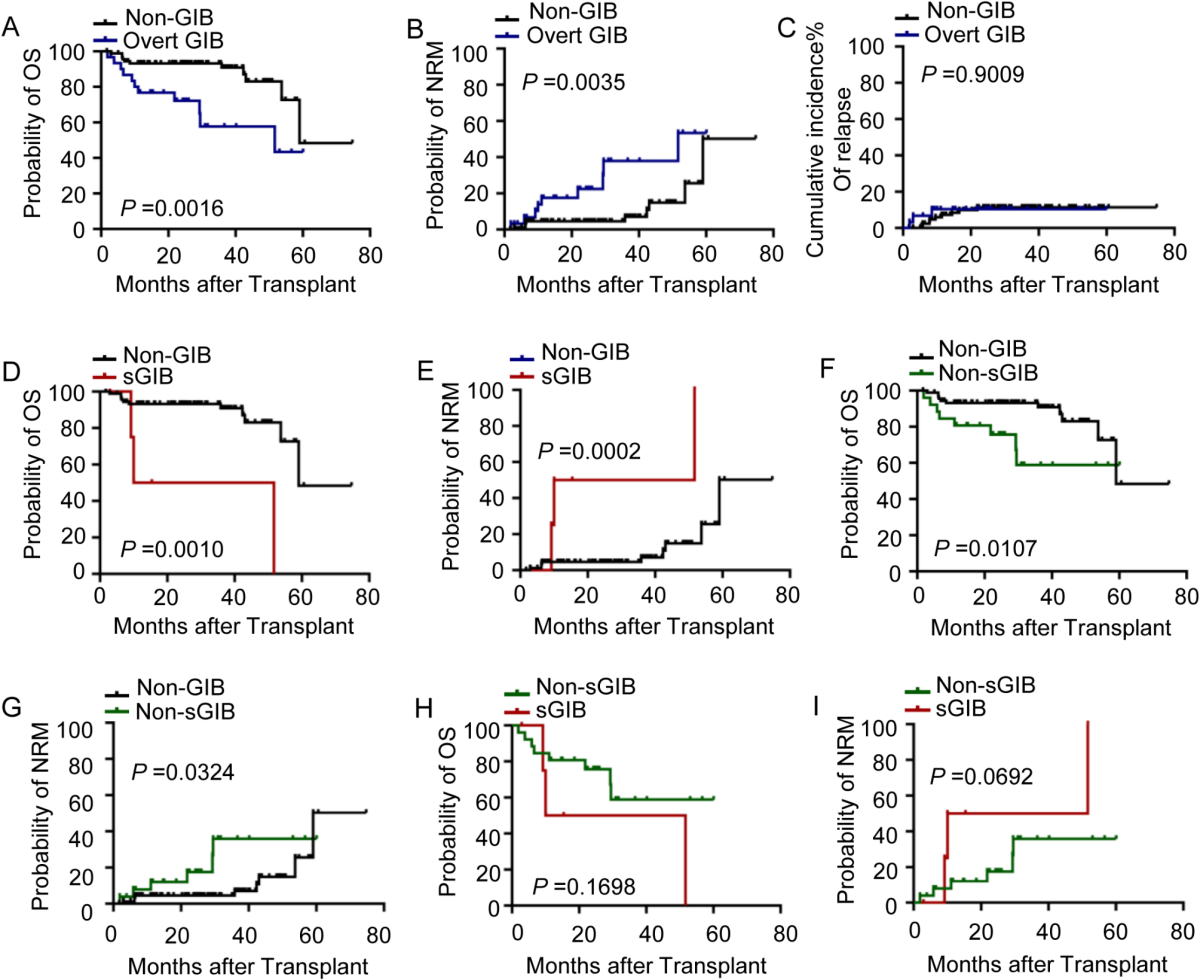
Clinical outcomes of patients with overt GIB after haplo-HSCT. A-C. Overall survival (**A**), non-relapse mortality (**B**) and incidence of relapse (**C**) for patients with or without GIB after haplo-HSCT. D-E. The impact of severe GIB on overall survival (**D**) and non-relapse mortality (**E**) after haplo-HSCT. F-G. The impact of non-severe GIB on overall survival (**F**) and non-relapse mortality (**G**) after haplo-HSCT. H-I. Comparison of overall survival (**H**) and non-relapse mortality (**I**) of patients with severe GIB and non-severe GIB. GIB, gastrointestinal bleeding; NRM, non-relapse mortality; OS, overall survival

The median follow-up period was 26.3 months (range, 1.7–74.8 months). The OS of patients with GIB were significantly reduced (*P* = 0.0016, Fig. 2.A), and the NRM was higher in these patients than in those without GIB (*P* = 0.0035, Fig. 2.B). There was no significant difference in the cumulative incidence of relapse between patients with GIB and the control group (*P* = 0.9009, Fig. 2.C). In subgroup analyses, it was observed that both severe GIB and non-severe GIB patients had significantly inferior OS (both *P* < 0.05, Fig. 2.D and F) and higher NRM (both *P* < 0.05, Fig. 2.E and G) when compared with non-GIB patients. However, OS and NRM showed no difference between patients with severe GIB and non-severe GIB (both *P* > 0.05, Fig. 2.H and I).

### Predictors for the occurrence of GIB

Patients with overt GIB had significantly higher cumulative incidences of III-IV gut aGvHD, TMA, CMV viremia and intestinal infection than those without GIB (Supplemental Fig. 2.A-D). To further identify the variables capable of predicting GIB after haplo-HSCT, univariate and multivariate analyses were carried out (Table 3). grade III-IV gut aGvHD, TMA, CMV viremia and intestinal infection were significantly related to the occurrence of GIB in the univariate analysis. In the multivariate analysis, grade III-IV gut aGvHD, TMA and CMV viremia retained their association with GIB.

**Table 3:** Risk factors for overt GIB after haplo-HSCT

Variables	Univariate			Multivariate		
	HR	95%	*P* value	HR	95%	*P* value
Age > 5.5 yr*	1.11	0.49–2.51	0.795			
Male	0.785	0.347–1.776	0.561			
ABO mismatch	0.93	0.41–2.12	0.879			
Donor-recipient sex mismatch	0.78	0.34–1.78	0.569			
Acute kidney injury	1.36	0.14–12.68	0.785			
Disseminated intravascular coagulation	0.66	0.058–7.61	0.744			
Hemorrhagic cystitis	0.62	0.25–1.52	0.298			
Sinusoidal obstruction syndrome	0.47	0.12–1.79	0.270			
cGVHD	0.585	0.225–2.221	0.552			
Grade III–IV gut aGvHD	17.50	1.95–156.49	0.010	19.21	1.78–207.79	0.015
Thrombotic microangiopathy	6.41	1.73–2.38	0.005	4.24	1.01–17.76	0.048
CMV viremia	4.45	1.74–11.38	0.002	4.25	1.38–10.74	0.010
Intestinal infection	3.65	1.23–10.78	0.019	1.99	0.56–7.03	0.284

*Notes* aGVHD, acute graft versus host disease; CMV, cytomegalovirus; cGVHD, chronic graft versus host disease; *: the median age of overt GIB patients

## Discussion

GIB in children can lead to substantial morbidity and mortality, especially in cases of severe GIB [14, 22, 23]. As haplo-HSCT is being used with increasing frequency for pediatric patients [24–26], systematic investigations of GIB are needed.

The published prevalence of overt GIB ranges from 5.9 to 39%, and severe GIB can constitute between 11.8% and 54.3% [14, 16, 27, 28]. In our study, our results indicated that overt GIB is a frequent complication in pediatric patients after haplo-HSCT (incidence of 25.2%). Fortunately, we found that 90.3% (28/31) of incidences of GIB in our study were either self-limited or controllable with supportive care only. Only 3 out of 31 (9.7%) patients experienced significant hemorrhage and required monitoring and management in an intensive care unit. While some studies have demonstrated that the severity of bleeding affects patient survival [16, 21], our results indicated that OS and NRM were not different between children with severe GIB and those with non-severe GIB.

There are reports that more than half of cases of intestinal bleeding occur within 100 days after transplantation [21, 27]. However, the timespan of GIB onset was relatively broad in our study, with GIB events occurring early after HSCT or more than a year after. Significantly, one case of bleeding was observed at 1275 days following HSCT, with the underlying etiology identified as colitis. However, we are uncertain whether the patient’s colitis is a complication of transplantation. In our study, single cause bleeding accounted for the majority followed by multifactorial bleeding (38.7%). Among these multiple bleeding causes, intestinal infection was second only to aGvHD. 5 kinds of pathogens were detected, including CMV (3 cases), norovirus (2 case), clostridium difficile (2 cases), salmonella (one case) and enteropathogenic Escherichia coli (one case).

In recent years, studies have revealed risk factors for hemorrhagic complications after HSCT, including severe thrombocytopenia, grade III–IV aGvHD, and TMA [14, 17, 28]. In addition, sinusoidal obstruction syndrome, acute kidney injury and viral hepatitis have been identified as causing an increased risk of GIB [16]. Similarly, we found grade III–IV aGvHD and TMA were independent risk factors for pediatric GIB following haplo-HSCT. We did not observe any cases of concurrent viral hepatitis and GIB, but CMV viremia was identified as an independent risk factor. At our center, the management of severe thrombocytopenia was timely, and therefore low platelet counts were not included in our risk analysis. In addition, we found that acute kidney injury and sinusoidal obstruction syndrome were not correlated with GIB after haplo-HSCT, potentially explained by the low number of events. In fact, GIB is one of the most common manifestations of GvHD. Since, GvHD not only directly induces the injury of gastrointestinal epithelium but also associated with CMV reactivation [29] and TMA [30].

Limitations of this study were the relatively small sample size and the retrospective design without a comparative group, which might be accompanied by some biases. Nevertheless, to the best of our knowledge, this is the first study to provide data regarding pediatric GIB after haplo-HSCT.

To conclude, our study shows that both severe and non-severe GIB after haplo-HSCT could decrease the OS and increase the NRM of pediatric patients. Furthermore, grade III–IV gut aGvHD, TMA and CMV viremia are risk factors for its development. In future research, prospective controlled trials and multicenter studies to understand the causes and optimal management of pediatric GIB will be essential.

### Electronic supplementary material

Below is the link to the electronic supplementary material.

Supplementary Material 1

## Data Availability

The datasets used and analyzed during the current study are available from the corresponding author on reasonable request.

## Abbreviations

aGvHD: Acute graft versus-host disease
allo-HSCT: Allogeneic hematopoietic stem cell transplantation
ATG: Anti-thymocyte globulin
cGvHD: Chronic graft versus host disease
CMV: Cytomegalovirus
DIC: Disseminated intravascular coagulation
GIB: Gastrointestinal bleeding
haplo-HSCT: Haploidentical hematopoietic stem cell transplantation
HLA: Human leukocyte antigen
NRM: Non-relapse mortality
OS: Overall survival
TMA: Thrombotic microangiopathy
