# Supplementary material for: Clinical characteristics and outcomes of overt gastrointestinal bleeding in children undergoing haploidentical hematopoietic stem cell transplantation: a single-center retrospective analysis
Source: BMC Pediatr. 2024 Jul 27;24:479. doi: 10.1186/s12887-024-04950-5 (PMC11282719; doi:10.1186/s12887-024-04950-5)
Supplement: Supplementary file 1 — Supplementary Material 1 [file 12887_2024_4950_MOESM1_ESM.docx]

Supplemental Figure 1


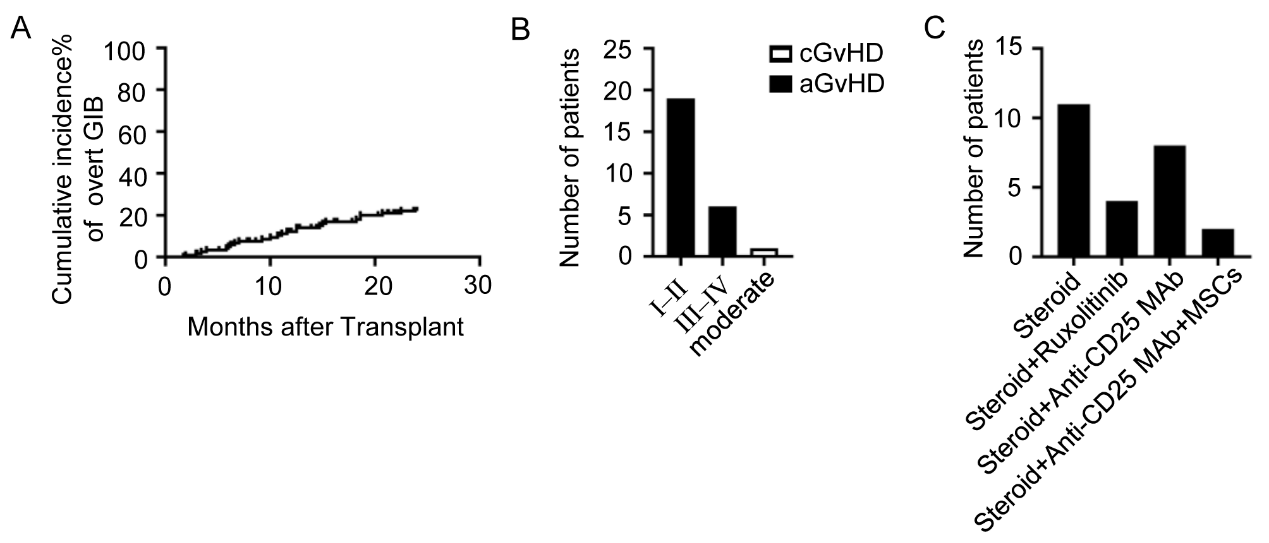


Supplemental Figure 1. A. Cumulative incidence of GIB at 2 years after haplo-HSCT. B. The distribution of GvHD grade among GIB patients. C. Treatment of GvHD among GIB patients. aGVHD, acute graft versus host disease; cGvHD, chronic graft versus host disease; GIB, gastrointestinal bleeding; MAb, Monoclonal antibody; MSCs, Mesenchymal stem cells.

Supplemental Figure 2


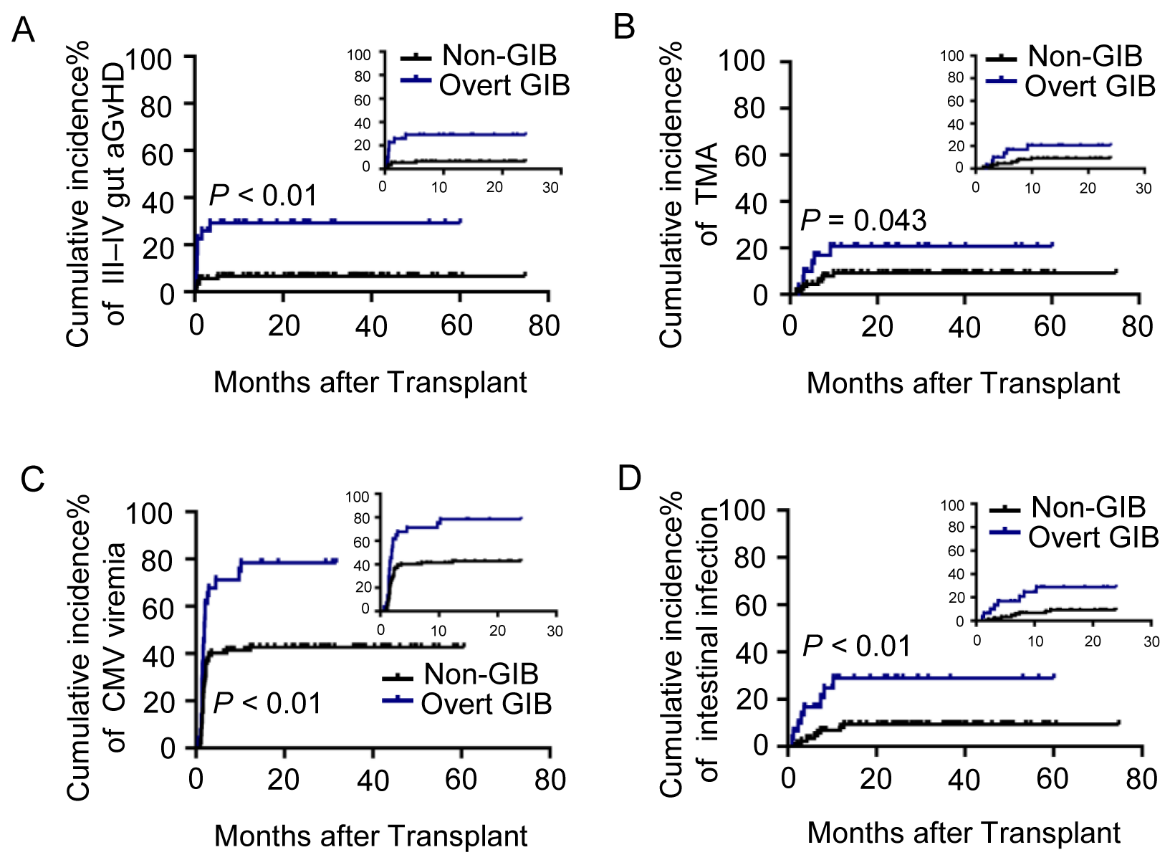


Supplemental Figure 2. A-D. Cumulative incidences of gut III–IV aGvHD (A), TMA (B), CMV viremia (C) and intestinal infection (D) among GIB patients and non-GIB patients.
